# STAFF PERSPECTIVE ON VIRTUAL TEAM-BASED CARE IN THE PROCESS OF TRANSITION TO LONG-TERM CARE

**DOI:** 10.1093/geroni/igae098.3170

**Published:** 2024-12-31

**Authors:** Lillian Hung, Chin Pang Ian Chan, Marie-Lee Yous, Denise Connelly, Karen Wong, Mariko Sakamoto, Paulina Santaella-Tafolla, Huini Ge

**Affiliations:** University of British Columbia, Vancouver, British Columbia, Canada; University of British Columbia, Vancouver, British Columbia, Canada; McMaster University, Hamilton, Ontario, Canada; Western University, London, Ontario, Canada; University of British Columbia, Vancouver, British Columbia, Canada; University of Victoria, Victoria, British Columbia, Canada; University of British Columbia, Vancouver, British Columbia, Canada; University of British Columbia, Vancouver, British Columbia, Canada

## Abstract

Transitioning from hospital to long-term care (LTC) is a vulnerable time for older patients and families. Successful care transitions require interdisciplinary team and cross-sectoral coordination, as well as engagement of patients and families in care planning. This study aimed to identify enablers and barriers to delivering virtual team-based care to support older adult care transitions from hospital to LTC. Patton’s utilization-focused approach informed the study design. Our research methods included semi-structured interviews, focus groups, and organizational policy reviews. The study involved 52 multidisciplinary team members, including nurses, physicians, rehabilitation practitioners, healthcare leaders, and older patients and family members. Thematic analysis identified key enablers (team engagement, training and support, and access to digital equipment) and barriers (technology infrastructure, resources, privacy and security). Our findings suggest that virtual team-based care is perceived by healthcare staff as acceptable, efficient, and cost-saving. Older persons and their families have complex care needs, and prioritizing their involvement in virtual team-based care planning is key to addressing barriers in the process of care transition success. Clear policies and guidelines are needed to support the integration of virtual team-based care into practice to optimize support to families and patient health outcomes. Future research should investigate effective strategies to include older persons and families through virtual team-based care planning technology.

